# Distribution of Young's Modulus in Porcine Corneas after Riboflavin/UVA-Induced Collagen Cross-Linking as Measured by Atomic Force Microscopy

**DOI:** 10.1371/journal.pone.0088186

**Published:** 2014-01-31

**Authors:** Jan Seifert, Christian M. Hammer, Johannes Rheinlaender, Saadettin Sel, Michael Scholz, Friedrich Paulsen, Tilman E. Schäffer

**Affiliations:** 1 Department of Applied Physics and LISA+, Eberhard-Karls-University, Tübingen, Germany; 2 Department of Anatomy II, Friedrich-Alexander-University, Erlangen, Germany; 3 Department of Ophthalmology, Ruprecht-Karls-University, Heidelberg, Germany; University of Reading, United Kingdom

## Abstract

Riboflavin/UVA-induced corneal collagen cross-linking has become an effective clinical application to treat keratoconus and other ectatic disorders of the cornea. Its beneficial effects are attributed to a marked stiffening of the unphysiologically weak stroma. Previous studies located the stiffening effect predominantly within the anterior cornea. In this study, we present an atomic force microscopy-derived analysis of the depth-dependent distribution of the Young's modulus with a depth resolution of 5 µm in 8 cross-linked porcine corneas and 8 contralateral controls. Sagittal cryosections were fabricated from every specimen and subjected to force mapping. The mean stromal depth of the zone with effective cross-linking was found to be 219±67 µm. Within this cross-linked zone, the mean Young's modulus declined from 49±18 kPa at the corneal surface to 46±17 kPa, 33±11 kPa, 17±5 kPa, 10±4 kPa and 10±4 kPa at stromal depth intervals of 0–50 µm, 50–100 µm, 100–150 µm, 150–200 µm and 200–250 µm, respectively. This corresponded to a stiffening by a factor of 8.1 (corneal surface), 7.6 (0–50 µm), 5.4 (50–100 µm), 3.0 (100–150 µm), 1.6 (150–200 µm), and 1.5 (200–250 µm), when compared to the Young's modulus of the posterior 100 µm. The mean Young's modulus within the cross-linked zone was 20±8 kPa (2.9-fold stiffening), while it was 11±4 kPa (1.7-fold stiffening) for the entire stroma. Both values were significantly distinct from the mean Young's modulus obtained from the posterior 100 µm of the cross-linked corneas and from the contralateral controls. In conclusion, we were able to specify the depth-dependent distribution of the stiffening effect elicited by standard collagen cross-linking in porcine corneas. Apart from determining the depth of the zone with effective corneal cross-linking, we also developed a method that allows for atomic force microscopy-based measurements of gradients of Young's modulus in soft tissues in general.

## Introduction

Keratoconus and other forms of corneal ectasia represent refractive pathologies of the eye characterized by a marked protrusion (i.e. outward bulging) of the cornea [1], [2], [3], [4]. This leads to pronounced visual impairment and is often associated with a significant deterioration of the patients' quality of life [5], [6]. In the majority of cases, these conditions are caused by an unphysiologically weak corneal stroma [4], [7], [8], [9]. Riboflavin/UVA-induced corneal collagen cross-linking (CXL) has become a widespread and effective clinical application to increase corneal stability [10], [11], [12], [13]. It has been demonstrated to stop the progression of keratoconus and to even improve visual acuity in keratoconus patients [14], [15], [16], [17]. Apart from that, it is potent enough to ameliorate or partially remedy ectatic complications after laser in situ keratomileusis (LASIK) [18]. To date, the exact molecular processes involved in CXL are largely unknown, although covalent bond formation due to UVA-induced radical ions and singlet oxygen seems to play a central role [19], [20], [21], [22]. It has been shown, however, that classical CXL predominantly strengthens the anterior stroma, while having no significant effect on the posterior cornea [23], [24], [25]. Attempts have been made to assess the maximum depth of effective CXL and depth-dependent CXL distribution [26]. To our knowledge, the only measurements available at present allowing for a deduction of biomechanical depth profiles throughout the entire stroma stem from confocal Brillouin microscopy [24], [27].

In this study, atomic force microscopy (AFM) nanoindentation was successfully used to create depth-dependent profiles of the Young's modulus (YM) with a depth resolution of 5 µm in porcine corneas after standard CXL. This method allowed identifying the depth of the zone with effective cross-linking (CXL zone). The approach presented here should be of value for the measurement of YM profiles not only in human or animal eyes, but also in other tissues.

## Materials and Methods

### Tissue preparation and CXL

**Figure 1:**
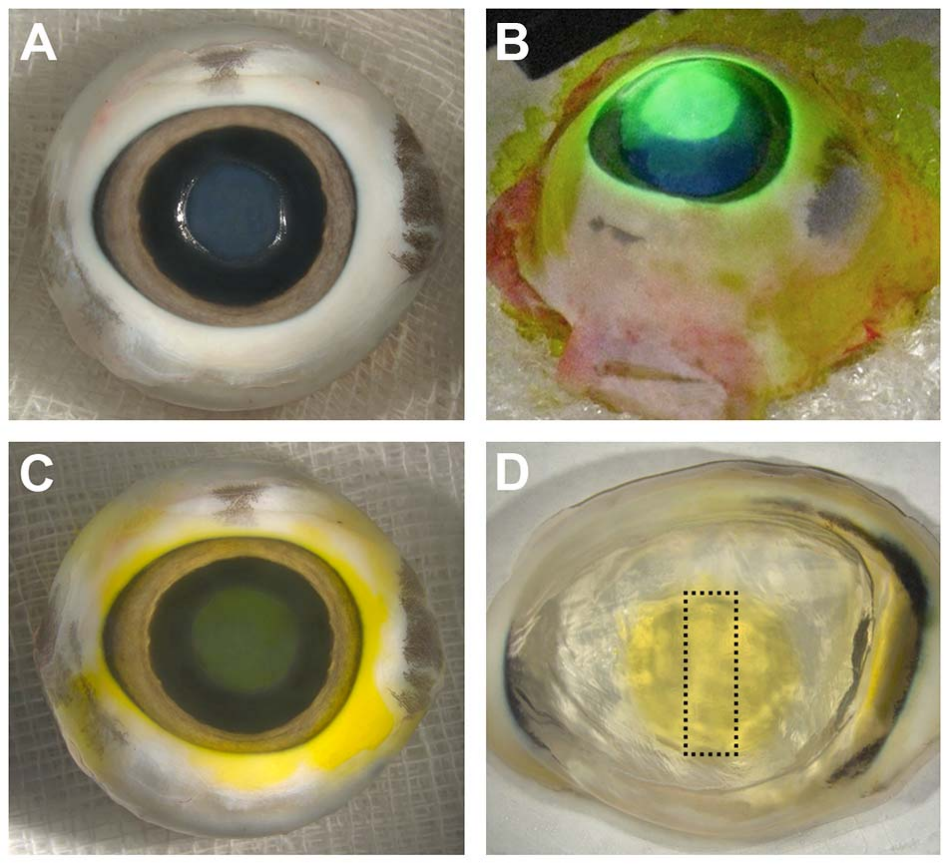
CXL procedure and sample preparation. Enucleated porcine eyes with centrally debrided corneal epithelium. Temporal side on the left, nasal side on the right. Superior aspect facing upwards, inferior aspect downwards. Note the markedly steeper curvature of the corneal margin on the temporal side (left). **A**) Prior to administration of riboflavin. Abraded area in the corneal center is clearly visible. **B**) UVA irradiation of riboflavin-saturated cornea. Note the pronounced fluorescence of riboflavin. **C**) After CXL. Note the marked yellow staining of the cornea within the abraded area due to stromal saturation with riboflavin. **D**) Excised corneoscleral ring. Conspicuous yellow staining of the cross-linked area. The dotted rectangle indicates shape and superior-inferior orientation of the dissected tissue specimen used for cryosectioning and AFM analysis.

Eight freshly enucleated and non-scalded pairs of porcine eyes (*n* = 16) were obtained from the local abattoir (Unifleisch GmbH & CoKG, Erlangen, Germany) and processed within 6 hours. Animal slaughter was performed in an approved facility and in accordance with the German national regulations. The eyes were transported and stored short-term in phosphate buffered saline (PBS) on ice. Only intact eyes with clear and unspoiled corneas were used in this study. After removal of extra-ocular muscle and fat tissue, the globes were briefly washed in PBS. Then, the corneal epithelium was abraded centrally in a ∼7 mm diameter zone with a hockey knife (blunt knife shaped like a hockey stick – standard tool used by ophthalmologists to remove corneal epithelium) to expose the corneal stroma (Figure 1A). A solution containing 0.1% riboflavin and 20% dextran (MedioCross®D, Medio-Haus Medizinprodukte GmbH, Kiel, Germany) was instilled dropwise onto the abraded corneas every 5 minutes for 30 minutes to saturate the stroma with the photosensitizer riboflavin (vitamin B2). After that, one eye out of every pair (*n* = 8) was exposed to UVA light emitted from a custom-built light source for 30 minutes (Figure 1B). The wavelength of the UVA diode chosen was 370 nm, which represents the absorption maximum of riboflavin. The administered irradiance was set to 3 mW/cm^2^, representing a fluence of 5.4 J/cm^2^ after 30 minutes of exposure. Wavelength and irradiance were in accordance with standard clinical procedures (Dresden protocol) [14], [15]. Before each treatment, irradiance was controlled with a calibrated power meter (LaserMate-Q™, Coherent GmbH, Dieburg, Germany) at a distance of 3 cm, which also represented the working distance in the experimental setup. Throughout the entire period of irradiation, riboflavin solution was instilled dropwise every 5 minutes, which resulted in a marked yellow staining of the exposed stroma (Figure 1C). The contralateral eyes (*n* = 8) served as non-cross-linked controls and were not exposed to UVA light, but otherwise treated identically. Throughout the entire period of riboflavin administration (and UVA irradiation) every porcine eye remained connected to a saline reservoir hanging 50 cm above the globe via an intravitreal cannula. This way, the intraocular pressure was standardized and ocular hypotony avoided. After cessation of UVA exposure, corneoscleral rings were dissected from the eyes and a superior-inferiorly oriented tissue sample measuring approximately 5×2×1 mm was prepared with a razor blade from every specimen (Figure 1D). In 4 specimens of every treatment group, this step was performed in PBS to minimize compression and shear forces during preparation (“PBS group”). In the remaining 4 specimens of every treatment group, this step was performed outside any fluids to minimize stromal hydration (“DRY group”). Then, the tissue samples were embedded in Tissue Tek® O.C.T. Compound (Sakura Finetek Germany GmbH, Staufen, Germany) using Tissue Tek® vinyl cryomolds measuring 15×15×5 mm and subsequently quick-frozen in nitrogen-cooled 2-methylbutane (Uvasol®, Merck KGaA, Darmstadt, Germany). Unfixed sagittal cryosections with a thickness of 16 µm were fabricated from every sample, mounted on Histobond®+ adhesion microscope slides (Paul Marienfeld GmbH & CoKG, Lauda-Königshofen, Germany) and kept frozen until AFM analysis. Detailed AFM analysis was performed on one cryosection per specimen after microscopical assessment of section quality.

### Atomic force microscopy

**Figure 2:**
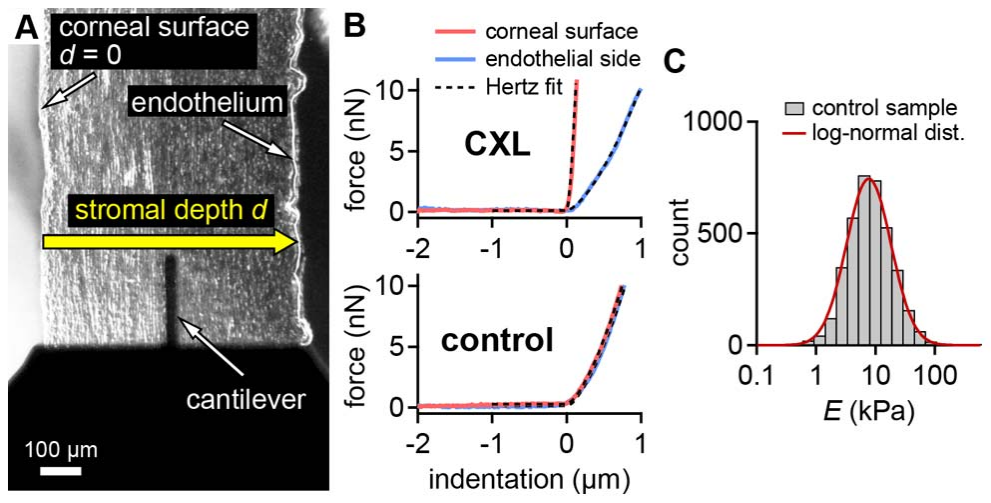
AFM-based creation of stromal YM profiles. **A**) Optical image of a cross-linked cornea section in the AFM setup. Force-indentation-curves were recorded across the stroma at an increasing stromal depth *d*. **B**) Representative force-indentation-curves at the corneal surface and at the endothelial side of CXL and control samples. A larger slope in the region of positive indentations represents a larger YM of the sample at the measured location. **C**) Representative stromal distribution of the local YM in non-cross-linked control samples, gained from force-indentation measurements across the stroma as described in A). The local YM follows a log-normal distribution.

The stromal distribution of the YM was measured using a commercial AFM setup (MFP3D-BIO, Asylum Research, Santa Barbara, CA) with a single sphere-tip cantilever (FM-M-SPL, Nanoworld, Neuchâtel, Switzerland, 980 nm tip radius) [28] in force-mapping mode [29]. The cantilever's spring constant was determined as 4.01 N/m with the thermal noise method [30]. Measurements were performed at room temperature in PBS, which was dripped onto the cryosections right after thawing. In each CXL and control section, force-indentation-curves were recorded across the stroma at an increasing stromal depth (*d*) in steps of 5 µm (Figure 2A). At each depth, 30 force-indentation-curves were recorded, spaced apart perpendicular to the corneal surface within a width of 90 µm. This was achieved by successively recording force maps with a scan size of 90×90 µm^2^, starting from the corneal surface, while the scanner was manually moved by 90 µm towards the endothelium after each force map. The local YM was calculated from each force-indentation-curve by least-squares fitting the spherical Hertz-model [31]:
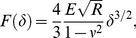
(1)where *F* is the measured force, *E* is the local YM, *R* is the cantilever's tip radius, *ν* is the Poisson's ratio of the sample (assumed as 0.5 for an incompressible material), and *δ* is the sample indentation. A larger slope in the force-indentation-curves (Figure 2B) indicates a larger local YM. By averaging the 30 values measured at each stromal depth, depth-dependent YM profiles with a depth resolution of 5 µm were obtained across the stroma. Averaging was done on the log-transformed values of *E* (giving the geometric average), because the stromal YM followed a log-normal distribution (see Results). Central corneal pachymetry was performed on every sectioned specimen using the optical unit of the atomic force microscope.

### Data analysis

The YM profiles of cross-linked corneas were fit by the exponential function
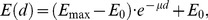
(2)where *d* is the stromal depth, *E*_0_ is the YM at the endothelial side, *E*_max_ is the YM at the corneal surface, and *μ* is the stiffening coefficient. Fitting was done using the method of least squares. To account for the log-normal distribution of *E* (see Results), the log-transformed fit function and the log-transformed values of *E*, were used for fitting. The depth of the CXL zone was defined as the stromal depth where the fit function *E*(*d*) reached 140% of *E*_0_. This corresponds to an 1.4-fold stiffening compared to the endothelial side.

Cumulated data from multiple samples are presented as arithmetic mean ± standard deviation (SD). The results were tested for significance using two-factor ANOVA (factor 1: CXL/control; factor 2: region of interest). Pairwise comparisons were done using Tukey HSD test. Only results with *p*-values below 0.05 were considered significantly different.

## Results

**Figure 3:**
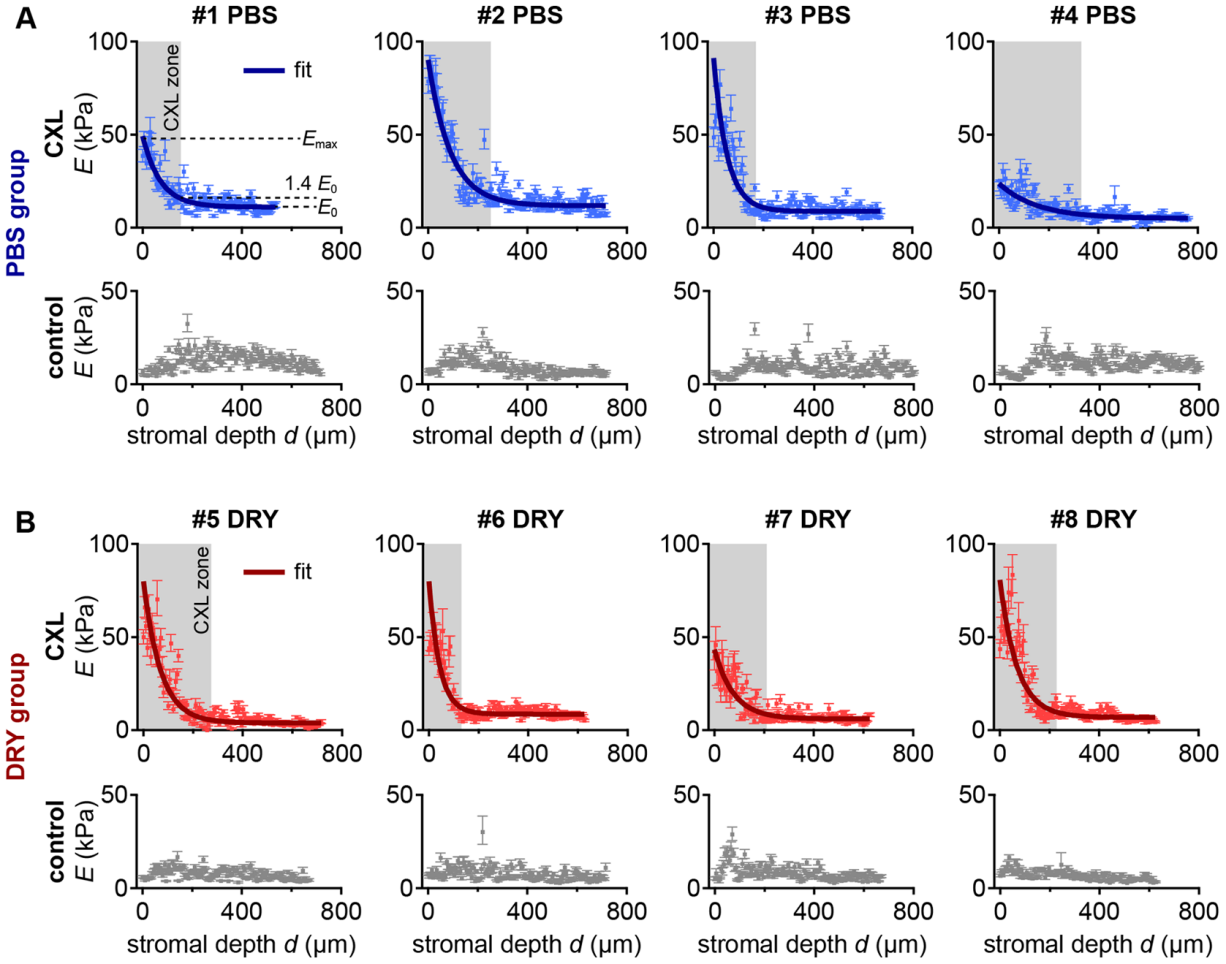
YM profiles across CXL and control corneas. A) Corneas dissected in PBS. B) Corneas dissected without any fluid (“DRY”). An increasing YM toward the corneal surface (*d* = 0) was found in the CXL samples. An exponential function (Equation 2) was fit to the YM profiles of CXL samples, giving *E*_max_ (YM at the corneal surface) and *E*_0_ (YM at the endothelial side). The CXL zone of effective cross-linking was defined as the zone where the YM of the fit exceeded 1.4 *E*_0_. Error bars: geometric standard error interval.

In all non-cross-linked control eyes, the YM followed a log-normal distribution (Figure 2C). Such a distribution has also been found for other types of tissue [32], [33], [34]. The YM profiles across the stroma showed no significant stiffening for both the PBS preparation group (Figure 3A “control”) and the DRY preparation group (Figure 3B “control”). The mean YM over the entire stroma was 9.4±1.7 kPa in the control corneas of the PBS group and 7.0±0.4 kPa in those of the DRY group. This difference was statistically significant (*p*<0.05), most likely due to stromal swelling in the PBS group. Calculation of the mean YMs using all 8 control samples yielded 8.2±1.7 kPa.

**Figure 4:**
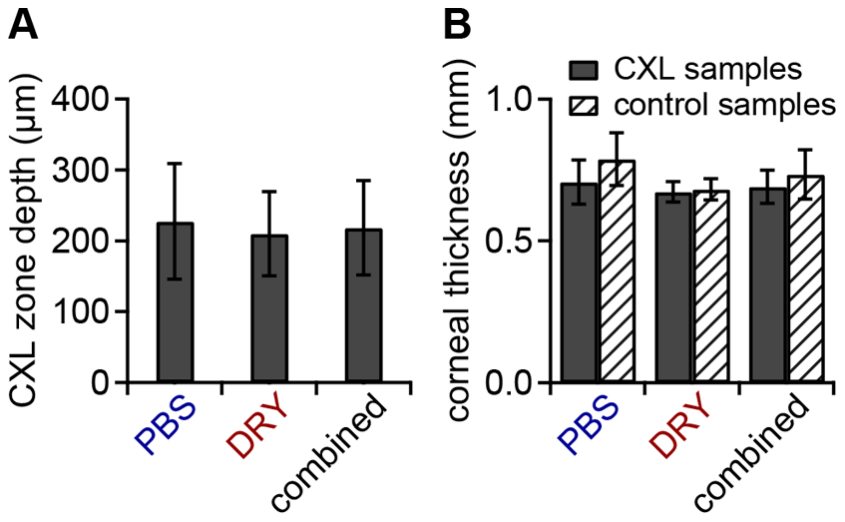
Depth of the CXL zone and corneal thickness. Pachymetry readings were executed centrally on every sectioned specimen using the optical unit of the atomic force microscope. **A**) Mean stromal depth of the CXL zone for the PBS group, the DRY group, and the combined groups. **B**) Mean corneal thickness at the location of the profile measurement for the PBS group, the DRY group, and the combined groups. No significant difference in corneal thickness was found with respect to preparation type or cross-linking status. Error bars indicate the standard deviation.

In every cross-linked sample examined, the YM was greatest at the corneal surface and declined markedly within the anterior 200 µm (Figure 3A “CXL” and B “CXL”). The measured YM profiles were well fit by an exponential function (Equation 2). The mean depth of the CXL zone with effective cross-linking was found at 227±82 µm (PBS group) and 210±59 µm (DRY group) (Figure 4A, Table 1). The mean thickness of the whole cornea was around 700 µm–800 µm for the CXL and control samples (Figure 4B). No significant difference in corneal thickness was found with respect to preparation type or cross-linking status.

**Table 1:** Stromal depth of the cross-linked (CXL) zone.

PBS group		DRY group		Combined	
**#1 PBS:**	154 µm	**#5 DRY:**	272 µm	-	
**#2 PBS:**	252 µm	**#6 DRY:**	130 µm	-	
**#3 PBS:**	171 µm	**#7 DRY:**	210 µm	-	
**#4 PBS:**	331 µm	**#8 DRY:**	228 µm	-	
**Mean ± SD:**	**227±82 µm**	**Mean ± SD:**	**210±59 µm**	**Mean ± SD:**	**219±67 µm**

CXL zone depth corresponds to the stromal depth exhibiting 1.4-fold stiffening.

The anterior 400 µm of the YM profiles were divided into consecutive stromal depth intervals of 50 µm. For each specimen, the average YM was calculated in every depth interval. With increasing stromal depth, this yielded the following mean YM over all samples: 46±17 kPa (0–50 µm), 33±11 kPa (50–100 µm), 17±5 kPa (100–150 µm), 10±4 kPa (150–200 µm), 10±4 kPa (200–250 µm), 10±4 kPa (250–300 µm), 10±3 kPa (300–350 µm) and 8.9±3.0 kPa (350–400 µm). Individual values for each specimen as well as the cumulated mean YM and the corresponding stiffening factors are listed in Table 2 and Table 3 for the PBS group and the DRY group, respectively. Mean YMs and corresponding stiffening factors within the stromal depth intervals of both preparation groups combined are given in Table 4. As the YM plateaued within the posterior 100 µm of each section and showed no significant difference to the corresponding YM of the control sections, this region was considered unaffected by CXL and served as a reference for comparison. Stiffening factors were determined by division of the YM in a region of interest by the YM of the posterior 100 µm. The mean YM over all CXL samples in this region was 7.0±2.7 kPa.

**Table 2:** Young's modulus *E* (kPa) within stromal depth intervals in the PBS group.

Stromal depth	#1 PBS	#2 PBS	#3 PBS	#4 PBS	Mean ± SD	Stiffening factor
**0 µm–50 µm**	40	75	54	20	**47±23**	5.3±1.8
**50 µm–100 µm**	25	51	38	18	**33±14**	3.8±1.1
**100 µm–150 µm**	18	27	18	15	**19±5**	2.3±0.4
**150 µm–200 µm**	13	19	9.1	9.9	**13±4**	1.5±0.3
**200 µm–250 µm**	13	20	7.7	8.7	**12±5**	1.4±0.4
**250 µm–300 µm**	13	18	8.8	7.7	**12±5**	1.3±0.2
**300 µm–350 µm**	13	16	12	7.0	**12±4**	1.4±0.1
**350 µm–400 µm**	12	13	11	5.9	**10±3**	1.2±0.1
**Posterior 100 µm**	10	11	7.9	5.6	**8.6±2.3**	1.0±0.0

Averaged Young's modulus of each sample from the PBS group for stromal depth intervals of 50 µm. Mean Young's moduli across all PBS samples and mean stiffening factors were calculated separately for each depth interval.

**Table 3:** Young's modulus *E* (kPa) within stromal depth intervals in the DRY group.

Stromal depth	#5 DRY	#6 DRY	#7 DRY	#8 DRY	Mean ± SD	Stiffening factor
**0 µm–50 µm**	51	45	30	58	**46±12**	10±6
**50 µm–100 µm**	32	29	26	44	**33±8**	7.0±3.4
**100 µm–150 µm**	21	9.9	13	18	**16±5**	3.6±2.7
**150 µm–200 µm**	8.5	8.1	8.2	8.1	**8.2±0.2**	1.8±0.9
**200 µm–250 µm**	5.9	7.7	6.8	10	**7.6±1.7**	1.5±0.5
**250 µm–300 µm**	3.4	10	8.3	11	**8.2±3.4**	1.5±0.3
**300 µm–350 µm**	5.5	10	8.0	8.5	**8.0±1.9**	1.6±0.3
**350 µm–400 µm**	5.4	10	6.3	7.7	**7.4±2.1**	1.5±0.3
**Posterior 100 µm**	2.8	7.6	5.4	5.5	**5.3±1.9**	1.0±0.0

Averaged Young's modulus of each sample from the DRY group for stromal depth intervals of 50 µm. Mean Young's moduli across all DRY samples and mean stiffening factors were calculated separately for each depth interval.

**Table 4:** Mean Young's modulus *E* (kPa) and corresponding stiffening factor within stromal depth intervals of both groups (PBS and DRY) combined.

Stromal depth	Mean ± SD	Stiffening factor
**0 µm–50 µm**	**46±17**	7.6±4.7
**50 µm–100 µm**	**33±11**	5.4±2.9
**100 µm–150 µm**	**17±5**	3.0±1.9
**150 µm–200 µm**	**10±4**	1.6±0.6
**200 µm–250 µm**	**10±4**	1.5±0.4
**250 µm–300 µm**	**10±4**	1.4±0.3
**300 µm–350 µm**	**10±3**	1.5±0.2
**350 µm–400 µm**	**8.9±3.0**	1.3±0.3
**Posterior 100 µm**	**7.0±2.7**	1.0±0.0

**Figure 5:**
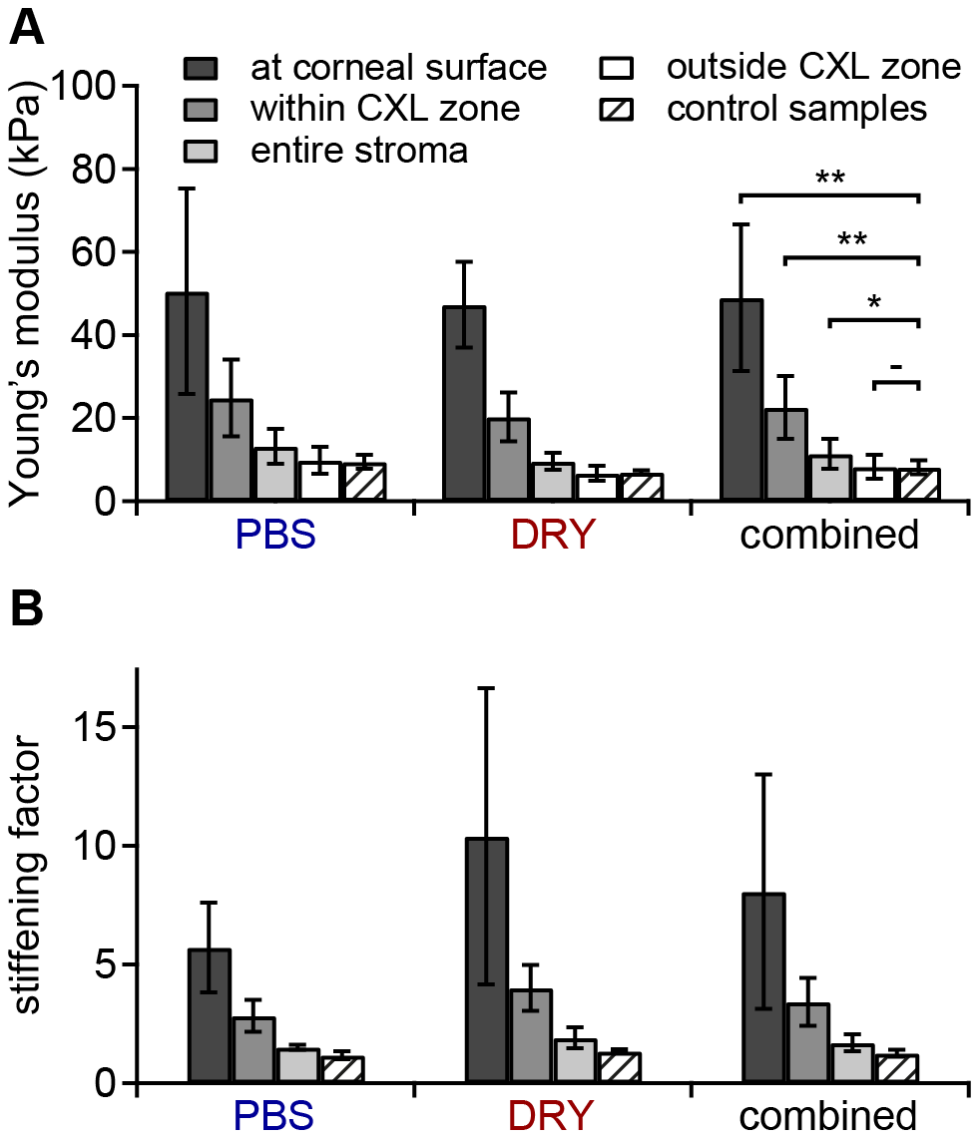
Young's moduli and stiffening factors. **A**) Mean YM at the corneal surface (anterior 25 µm), in the CXL zone, in the entire stroma, and outside the CXL zone. Control samples are included for comparison. Stars indicate a significant difference (* *p*<0.05, ** *p*<0.01, - not significant). **B**) Mean stiffening factors as determined by division of the YM of each sample in the region of interest (see A) by the YM of the posterior 100 µm. Error bars indicate the standard deviation.

Maximum stiffening in all CXL samples occurred at the corneal surface, where the mean YM was 49±18 kPa (Figure 5A, Table 5). This was significantly higher (*p*<0.01) than the mean YM at the posterior 100 µm. The mean stiffening factor at the corneal surface was 8.1 (Figure 5B, Table 5). In the CXL zone, the mean YM was 20±8 kPa (Figure 5A, Table 6), representing a statistically significant 2.9-fold stiffening (*p*<0.01). The mean YM in the entire stroma was 11±4 kPa (Figure 5A, Table 7), representing a significant 1.7-fold stiffening (*p*<0.05). Individual values for each sample and means are given in Table 5, Table 6 and Table 7 for the corneal surface, the CXL zone and the entire cornea, respectively.

**Table 5:** Young's moduli *E* (kPa) and stiffening factors at the corneal surface.

Sample	*E* (kPa)	Stiffening factor
#1 PBS	41	4.1
#2 PBS	81	7.4
#3 PBS	58	7.3
#4 PBS	23	4.0
**Mean±SD #1-#4**	**51±25**	**5.7±1.9**
#5 DRY	55	19
#6 DRY	48	6.3
#7 DRY	33	6.0
#8 DRY	54	9.9
**Mean±SD #5-#8**	**47±10**	**10±6**
**Mean±SD #1-#8**	**49±18**	**8.1±4.9**

Averaged Young's modulus and stiffening factor of each sample from the PBS and DRY groups at the corneal surface (anterior 25 µm). Mean Young's moduli and mean stiffening factors were calculated for each preparation group and combined.

**Table 6:** Young's moduli *E* (kPa) and stiffening factors across the cross-linked (CXL) zone.

Sample	*E* (kPa)	Stiffening factor
#1 PBS	25	2.6
#2 PBS	35	3.2
#3 PBS	21	2.6
#4 PBS	12	2.1
**Mean±SD #1-#4**	**23±10**	**2.6±0.5**
#5 DRY	12	4.2
#6 DRY	20	2.6
#7 DRY	14	2.6
#8 DRY	18	3.3
**Mean±SD #5-#8**	**16±4**	**3.2±0.7**
**Mean±SD #1-#8**	**20±8**	**2.9±0.7**

Averaged Young's modulus and stiffening factor of each sample from the PBS and DRY groups across the CXL zone. Mean Young's moduli and mean stiffening factors were calculated for each preparation group and combined.

**Table 7:** Young's moduli *E* (kPa) and stiffening factors across the entire stroma.

Sample	*E* (kPa)	Stiffening factor
#1 PBS	15	1.5
#2 PBS	18	1.6
#3 PBS	12	1.5
#4 PBS	7.8	1.4
**Mean±SD #1-#4**	**13±4**	**1.5±0.1**
#5 DRY	7	2.5
#6 DRY	11	1.5
#7 DRY	8.9	1.6
#\RY	11	2.0
**Mean±SD #5-#8**	**9.6±2.0**	**1.9±0.4**
**Mean±SD #1-#8**	**11±4**	**1.7±0.4**

Averaged Young's modulus and stiffening factor of each sample from the PBS and DRY groups across entire stroma. Mean Young's moduli and mean stiffening factors were calculated for each preparation group and combined.

## Discussion

Combined application of riboflavin and UVA during corneal CXL induces the formation of additional covalent bonds within the corneal stroma. This involves collagen fibers as well as corneal proteoglycan core proteins [35]. Apart from illumination time, the efficacy of this process should theoretically be dependent on the local concentration of riboflavin and the intensity of UVA irradiation present at the site of CXL [36]. As for standard CXL, both parameters have been demonstrated to decrease exponentially with increasing stromal depth [36], [37], although the stromal distribution of riboflavin remains controversial [38], [39], [40]. Owing to the exponential decline of UVA irradiation (Lambert-Beer's law) an exponential reduction of CXL-induced corneal stiffening is expected in riboflavin-soaked corneas, assumed that the stiffening effect is proportional to the UVA intensity. This is utterly in line with the data presented here. To our knowledge, the present study is the first to empirically substantiate the exponential decrease of stromal YM after corneal CXL by a direct mechanical method of measurement. At a certain depth, UVA intensity and maybe also the local concentration of riboflavin should become too low for effective cross-linking. This is in agreement with previous research demonstrating significant stromal stiffening after CXL for the anterior cornea only [23], [24], [25]. These findings are corroborated by our data. We further determined the depth of the CXL zone, which we defined as the depth where the YM became 1.4× larger than that of the non-cross-linked endothelial side. The depth of the CXL zone was determined as 219±67 µm, which is in accordance with previous estimations by Chai *et al.* [26] and Schumacher *et al.* [36] who predicted the maximum depth of the CXL zone to be located between 200 µm and 300 µm. Stress-strain measurements published by Kohlhaas *et al.* [23] showed a significant stiffening after CXL of porcine and human corneas within the anterior 200 µm only. The adjacent 200 µm of corneal stroma did not show any significant stiffening by standard CXL in human and porcine eyes. Quite obviously, this constitutes a good match to the data presented here. Also the fact that we found no significant depth-dependent variation of the YM in the non-cross-linked porcine cornea is in line with Kohlhaas *et al.* [23]. In untreated human corneas, however, a gradient with a greater YM in the anterior stroma was detected by previous studies [23], [24], [25], [41]. Across the entire depth of the porcine corneas a mean stiffening factor of 1.7 was determined in the present study, which is perfectly in line with stress-strain measurements performed by Wollensak *et al.* [11] who found a factor of 1.8. However, a direct comparison of the YM values from this study with values gained from tensile tests such as stress-strain measurements is problematic: AFM compresses the sample on the nanometer scale, whereas tensile tests stretch the sample on a macroscopic scale (i.e. in a different direction and on a different length scale). Measurements with different techniques on the same type of tissue might therefore give different YM values.

One of the major drawbacks of the method employed in this study is the impossibility of *in vivo* measurements, owing to the necessity of cryosections. Recently, confocal Brillouin microscopy was introduced by Scarcelli *et al.* as a novel means of acquiring depth-dependent local micromechanical properties of human and animal corneas *in vivo* [24], [27]. The authors measured YM profiles that seem to be partially in conflict with our data and with the findings of other researchers. Kohlhaas *et al.* [23], Chai *et al.* [26] and Schumacher *et al.* [36] suggest a maximum stromal depth of the CXL zone between 200 µm and 300 µm. As opposed to this, the data of Scarcelli *et al.* [24] appear to yield values around 700 µm. It is up to future discussion, if and how these findings can be brought in alignment with the evidence presented by other research groups including ours. Probably, the utilization of fundamentally different methods of data generation may at least partially account for the discrepancies described.

The AFM measurements presented in this study were performed on sagittal cryosections. This may have caused an alteration and maybe partial distortion of the YM in native tissue for two reasons. Firstly, freezing the tissue and thawing it again for measurements has been demonstrated to change its inherent biomechanical properties in soft tissues other than cornea [42], [43]. Secondly, disturbing the three-dimensional integrity of the highly organized collagen lattices by severing the fibers during the cutting process is likely to have a profound impact on the performed measurements. Both factors may also contribute to the variation observed among the measurements. However, relative comparisons of AFM data are possible on cryosections, as already demonstrated [28]. Another limiting factor may be constituted by the spatial orientation of the indentation process. As the cryosections used were cut sagittally, the AFM indenter acted on the stroma in the frontal plane. This represented an orientation perpendicular to the physiological direction of force exerted on the central cornea by the intraocular pressure (sagittal plane). Yet, in the course of CXL, covalent bond formation within the corneal stroma is very likely to take place in three spatial dimensions. Hence, depth-dependent gradients of CXL efficacy should be detectable by nanoindentation on sagittal sections of the cornea with satisfying accuracy.

Stromal hydration and swelling is always an issue for corneal biomechanics in dissected specimens. The stromal tissue response to external forces depends on the hydration status, as indicated by biomechanical measurements after varying stromal hydration [44]. Additionally, tensile overstrain of collagen fiber lattices due to shear forces may also cause a distortion of biomechanical data. Both factors were addressed by dissecting one group of corneal specimens in PBS (reduction of shear forces, augmented hydration) and the other one outside any liquid (reduced hydration, higher risk of destructive shear forces). The PBS group showed a considerably lower degree of stiffening compared to the DRY group. Moreover, the measured YM profiles appeared to be more consistent in the DRY group, although a definitive statement is difficult to make due to the small sample size. As fiber overstrain due to shear forces during preparation obviously was not an issue for nanoindentation analysis, we recommend to avoid the use of any fluids for tissue processing between CXL and embedding.

## Conclusions

AFM nanoindentation had been applied before to determine spatial differences of the YM in cross-linked and non-cross-linked corneas [25], [45]. In these studies, single indentations at various locations were performed. This allowed for a gross comparison of the YM at the corneal surface and endothelial side, but provided no actual distribution of the YM across the stroma. The approach presented here yielded the first AFM-derived YM profiles across the full depth of the central cornea with a depth resolution of 5 µm. AFM allowed us to empirically confirm the exponentially declining nature of stromal stiffening after CXL in porcine corneas. Furthermore, it enabled us to estimate the depth of effective corneal cross-linking due to standard CXL. We anticipate that our method of measuring profiles of micromechanical properties in porcine corneas may also be applied to human corneas and other types of soft tissues.
